# Value of intraventricular dyssynchrony assessment by gated-SPECT myocardial perfusion imaging in the management of heart failure patients undergoing cardiac resynchronization therapy (VISION-CRT)

**DOI:** 10.1007/s12350-018-01589-5

**Published:** 2019-01-25

**Authors:** Amalia Peix, Ganesan Karthikeyan, Teresa Massardo, Mani Kalaivani, Chetan Patel, Luz M. Pabon, Amelia Jiménez-Heffernan, Erick Alexanderson, Sadaf Butt, Alka Kumar, Victor Marin, Claudio T. Mesquita, Olga Morozova, Diana Paez, Ernest V. Garcia

**Affiliations:** 1 Nuclear Medicine Department, Institute of Cardiology, 17 No. 702, Vedado, CP 10 400, La Habana, Cuba; 2 All India Institute of Medical Sciences, New Delhi, India; 3 Hospital Clínico Universidad de Chile, Santiago, Chile; 4 Fundación Valle del Lili, Cali, Colombia; 5 Hospital Juan Ramón Jiménez, Huelva, Spain; 6 Instituto Nacional de Cardiología Ignacio Chávez, Mexico, DF Mexico; 7 Oncology and Radiotherapy Institute (NORI), Islamabad, Pakistan; 8 Dr. B L Kapur Memorial Hospital, New Delhi, India; 9 Fundación Cardioinfantil, Bogotá, Colombia; 10 Hospital Universitario Antonio Pedro, Niteroi, Brazil; 11 Nuclear Medicine and Diagnostic Imaging Section, International Atomic Energy Agency, Vienna, Austria; 12 Emory University, Atlanta, GA USA

**Keywords:** Single-photon emission computed tomography, dyssynchrony, phase analysis, cardiac resynchronization therapy

## Abstract

**Background:**

Placing the left ventricular (LV) lead in a viable segment with the latest mechanical activation (vSOLA) may be associated with optimal cardiac resynchronization therapy (CRT) response. We assessed the role of gated SPECT myocardial perfusion imaging (gSPECT MPI) in predicting clinical outcomes at 6 months in patients submitted to CRT.

**Methods:**

Ten centers from 8 countries enrolled 195 consecutive patients. All underwent gSPECT MPI before and 6 months after CRT. The procedure was performed as per current guidelines, the operators being unaware of gSPECT MPI results. Regional LV dyssynchrony (Phase SD) and vSOLA were automatically determined using a 17 segment model. The lead was considered on-target if placed in vSOLA. The primary outcome was improvement in ≥1 of the following: ≥1 NYHA class, left ventricular ejection fraction (LVEF) by ≥5%, reduction in end-systolic volume by ≥15%, and ≥5 points in Minnesota Living With Heart Failure Questionnaire (MLHFQ).

**Results:**

Sixteen patients died before the follow-up gSPECT MPI. The primary outcome occurred in 152 out of 179 (84.9%) cases. Mean change in LV phase standard deviation (PSD) at 6 months was 10.5°. Baseline dyssynchrony was not associated with the primary outcome. However, change in LV PSD from baseline was associated with the primary outcome (OR 1.04, 95% CI 1.01-1.07, *P* = .007). Change in LV PSD had an AUC of 0.78 (0.66-0.90) for the primary outcome. Improvement in LV PSD of 4° resulted in the highest positive likelihood ratio of 7.4 for a favorable outcome. In 23% of the patients, the CRT lead was placed in the vSOLA, and in 42% in either this segment or in a segment within 10° of it. On-target lead placement was not significantly associated with the primary outcome (OR 1.53, 95% CI 0.71-3.28).

**Conclusion:**

LV dyssynchrony improvement by gSPECT MPI, but not on-target lead placement, predicts clinical outcomes in patients undergoing CRT.

**Electronic supplementary material:**

The online version of this article (10.1007/s12350-018-01589-5) contains supplementary material, which is available to authorized users.

## Introduction

Heart failure affects more than 15 million people worldwide and is growing globally at epidemic proportions, causing considerable increases in disability, mortality, and healthcare costs.^1^

As a consequence of the epidemiologic transition and advances in health care, as well as the aging of the population and the high prevalence of coronary artery disease (CAD), hypertension, obesity, and diabetes mellitus are increasing and will have a significant impact on the incidence of heart failure in low-and-middle income countries. Therefore, in a few years, the incidence and prevalence of heart failure may reach similar levels to those observed in high-income countries.^2^ Cardiac resynchronization therapy (CRT) can benefit some patients with end-stage heart failure, depressed left ventricular ejection fraction (LVEF) (<35%), and a wide QRS complex on the surface electrocardiogram (>120 milliseconds).^3^ However, these selection criteria are suboptimal, given that in previous CRT trials which used them, a significant percentage of patients (20-40%) did not benefit from CRT.^4^,^5^ It has been recognized that electrical dyssynchrony as determined by QRS duration may not necessarily represent real mechanical dyssynchrony and, therefore, not the best predictor of CRT response.^6^-^8^ Therefore, assessment of cardiac mechanical dyssynchrony is needed to more accurately select patients who would benefit more consistently from CRT. It has been shown that left ventricular mechanical dyssynchrony may be mandatory for the prediction of CRT response.^9^,^10^

Assessment of LV dyssynchrony has been approached with a number of imaging techniques, such as echocardiography with tissue Doppler imaging, strain imaging and more recently speckle-tracking; magnetic resonance imaging; gated blood pool ventriculography and gated single photon emission computed tomography (gSPECT).^11^-^17^

LV mechanical dyssynchrony, site of latest mechanical activation, and myocardial scarring are important parameters related to CRT response.^18^,^19^

Since many heart failure patients will undergo a gSPECT myocardial perfusion imaging (MPI) study as part of the work-up, added to the ventricular function information and the perfusion images to assess the presence, extent and location of myocardial scar or fibrotic tissue, these patients can benefit from the simple additional phase analysis to measure LV dyssynchrony. Although this knowledge may influence site selection for LV pacing lead placement, to date, there is no multicenter trial evidence to support it. Additional potential benefits for gSPECT MPI are its widespread availability, automation, and reproducibility and the ability to obtain data from already acquired gSPECT MPI studies.

Therefore, the International Atomic Energy Agency (IAEA) has sponsored a non-randomized, multicenter trial: “Value of intraventricular synchronism assessment by gated-SPECT myocardial perfusion imaging in the management of heart failure patients submitted to cardiac resynchronization therapy” (IAEA VISION-CRT). The aim of this trial is to improve the clinical response of heart failure patients by properly predicting response to CRT therapy and helping guide the optimal placement of the LV lead by using nuclear medicine techniques. Here, we report our primary findings.

## Methods

### Study Design

Ten centers from 8 countries (Brazil, Chile, Colombia, Cuba, India, Mexico, Pakistan, and Spain) participated in this prospective cohort study. The countries’ main investigators recorded all the data (clinical, CRT, gSPECT MPI, and follow-up information) in individual forms for each patient, and these data were collected by the central management center in the IAEA headquarters in Vienna.

In each participant center, the New York Heart Association (NYHA) class was assessed by a clinical cardiologist unaware of the imaging results as determined by the core lab. Minnesota Living With Heart Failure Questionnaire (MLHFQ)^®^ was administered by study personnel and change was assessed by the conventional 5-point criteria.

Emory University (USA) acted as a core lab for centralized gSPECT MPI reconstruction, processing and phase analysis. The core lab was blinded as to each patient’s clinical and CRT information. Statistical analysis of the acquired data was carried out by the All India Institute of Medical Sciences (India).

We aimed to assess the role of LV dyssynchrony and viability quantified from gSPECT MPI to predict clinical outcomes at six months in subjects undergoing CRT. Improved clinical response (primary outcome) was defined as at least one of the following 4 points at six months: improvement by at least 1 NYHA class, improvement of LVEF by ≥5%, reduction of end-systolic volume (ESV) by ≥15%, and improvement of LVEF by at least 5 points in MLHFQ.

The LV lead placement was not guided by gSPECT imaging data but by the conventional clinical practice of each participating site and then the results were compared as to whether it followed the SPECT recommendation (on-target) or not. We defined lead placement as on-target or recommended as in the study by Friehling et al.^20^ Thus, lead placement decisions were based on current standard international practice (posterolateral LV wall, depending on vein availability). Any other placement was defined as remote and not recommended as in any scarred segment.

The study was approved by the participant countries’ scientific councils and complies with the Declaration of Helsinki. Written informed consent was obtained from all participants and patient anonymity was maintained during data analysis.

### Patient Population

One hundred and ninety-eight patients underwent CRT, but complete data of clinical assessment, baseline core-lab SPECT and clinical six-month follow-up data were obtained in 195 patients.

**Figure 1:**
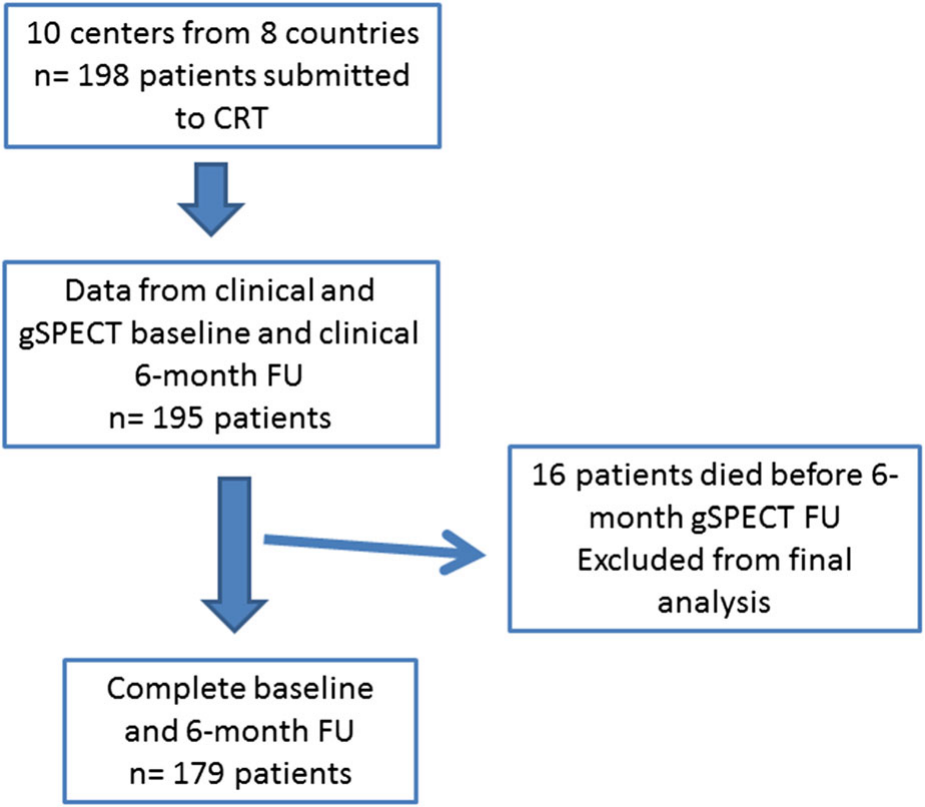
Study flow chart. *CRT*, cardiac resynchronization therapy; *FU*, follow-up; *gSPECT*, gated single-photon emission computed tomography

For the analysis, 195 sequential patients with gSPECT MPI who met the following inclusion criteria were included: stable patients over 18 years old with NYHA functional class II, III or ambulatory IV heart failure for at least three months before enrolment, despite receiving optimal tolerated medical therapy according to current guidelines; LVEF ≤ 35% from ischemic or non-ischemic causes, measured according to the usual procedure at the participating centre for inclusion, whereas LVEFs used for analysis came from nuclear core lab; intrinsic QRS duration of ≥120 ms, with morphology of left bundle branch block; sinus rhythm; written informed consent. Exclusion criteria were as follows: arrhythmias that prevented the gated acquisition; major coexisting illness affecting survival less than one year; right bundle branch block; pregnancy or breast-feeding; acute coronary syndromes, coronary artery bypass grafting or percutaneous coronary intervention in the last three months before enrolment and within six months of CRT implantation. These patients were studied by gSPECT MPI at rest within up to four weeks before the CRT implantation, and 6 ± 1 months after. As sixteen patients died between the baseline and follow-up period, 179 patients were included for the final analysis (Figure 1).

### SPECT Acquisition and Reconstruction

The SPECT scans were performed approximately 30 minutes post rest injection using 740-1110 MBq (20-30 mCi) of 99mTc-sestamibi or tetrofosmin. SPECT images were acquired on dual-headed cameras using 180° orbits and a standard resting protocol with either 8 or 16 frames ECG-gating according to standard current guidelines.^21^ All images were reconstructed using OSEM with 3 iterations and 10 subsets and filtered by a Butterworth filter, power 10, using a cut-off frequency of 0.3 cycles/mm. Reorientations into short-axis images were sent to ECTb4 (Emory Cardiac Toolbox, Atlanta, GA) for conventional automated processing of perfusion and function, including phase dyssynchrony analysis.

### Algorithm to Guide LV Lead Position Placement

Algorithms for measuring LV dyssynchrony^16^ and for guiding optimal LV lead position^18^,^20^ have been described in detail elsewhere. Briefly, a 3D sample distribution of maximum counts along the LV myocardial wall is extracted from each of the gated LV short axis data sets. A one-dimensional first-harmonic Fourier approximation is applied to the count variation over time for each myocardial segment, generating a 3D phase distribution that describes the timing of LV regional mechanical activation (onset of mechanical contraction) over the entire R-R cycle. The most clinically relevant dyssynchrony index derived is the phase standard deviation (PSD).^16^ The normal values have been published and validated.^16^,^22^ The technique is fully automated, has effective temporal resolution of 15 ms for a heart rate of 60/min, inter and intra-observer reproducibility of 99%,^23^ high repeatability,^24^ good robustness with camera types,^25^ tracer dose,^26^ heart rate and perfusion defects.^27^ The LV perfusion distribution voxels with less than 50% of the LV maximum voxel counts were defined as non-viable. The average onset of regional mechanical contraction (myocardial activation) is determined as the average phase for each of the 17 LV segments. Regional myocardial activation and viability are then combined to automatically identify the location of the viable segment with the latest mechanical activation (vSOLA) as the location of the optimal LV lead position.^20^,^28^,^29^ Other viable segments with average phases within 10° of the vSOLA were also automatically identified as potential candidates for lead placement and labeled as acceptable segments. Segments with phases wider than 10° of the vSOLA were not considered as candidates for lead implantation.

The position of the LV lead post-implantation was determined from postero-anterior and lateral chest radiography and localized on a 17-segment polar map of the LV by an electrophysiologist blinded to gSPECT MPI findings.

### Statistical Analysis

For power analysis, we assumed that dyssynchrony by imaging will be present in approximately two-thirds of any patient population submitted for CRT. From previous studies, we estimated that 75% of those who have dyssynchrony on imaging will demonstrate a clinical response. Assuming that the likelihood of a favorable clinical response is reduced by 30% (to about 53%) among those who do not demonstrate dyssynchrony on imaging, we estimated that a sample size of about 180 patients would be sufficient for a power of 80% at a two-tailed *α* of 0.05%.

Left ventricular dyssynchrony was defined as left ventricular phase histogram standard deviation (PSD) >43°,^30^ as determined by gSPECT MPI. The primary analysis was to determine the difference in the proportion of patients showing a favorable clinical response depending on the presence or absence of dyssynchrony. We determined the independent predictive value of LV dyssynchrony on the primary outcome after adjustment for other important prognostic variables in a logistic regression model. We adjusted our model for all the prognostically important baseline variables (which were not collinear). The variables adjusted for were, age, gender, QRS width, NYHA class, LVESV and LVEF. In exploratory analyses, we assessed the predictive value of improvement in dyssynchrony at six months as a predictor of the primary outcome. We calculated area under the receiver-operating curves (ROC) for both LV dyssynchrony at baseline and the improvement in dyssynchrony after CRT for predicting the primary outcome.

Lead position as recorded at the time of device implantation was categorized as on-target if it was placed in the vSOLA as identified on the SPECT study. It was considered acceptable if it was placed in segments within 10° of the last viable contracting segment. We assessed the reduction in LV dyssynchrony with lead position and determined its independent predictive value in predicting the primary outcome or its components. All analyses were performed using Stata 13 (College Station, Texas). We considered a *P* value <.05 to be statistically significant.

## Results

Baseline patients’ clinical characteristics are presented in Table 1. Only one patient was paced in apex, and seven patients were paced in segments deemed non-viable (by the established definition that any myocardial pixel <50% of maximum LV uptake is non-viable). QRS interval mean duration corresponded to 158.7 ± 25.4 ms, and LV PSD histogram to 54.4 ± 20.9°. There was no correlation between the two variables (*r* = .028; *P* = .698). Changes in the measurements of the primary endpoints are presented in Table 2. There were significant improvements in NYHA class, LVEF, MLHF score and LVESV. Fifty out of 195 (25.6%) patients had an infarct size >35% of LV.

**Table 1:** Baseline clinical characteristics of patients

Variable	N = 195
Age, years	60 (11)
Females, N (%)	74 (38)
Height, cm	164 (11)
Weight, kg	71 (15)
Ethnicity	
Hispanic	102 (52)
Asian Indian	38 (19)
Caucasian	26 (13)
African	20 (10)
History of CAD	60 (31)
Previous myocardial infarction	42 (22)
Previous revascularization	9 (5%)
Hypertension	111 (57)
Diabetes	50 (26)
Dyslipidemia	56 (29)
Smoking	38 (19)
Medical treatment	
Aspirin	96 (49)
Beta blockers	167 (85)
ACE inhibitors	118 (61)
ARBs	51 (26)
Diuretics	160 (82)
Statins	74 (38)

Age, height and weight are expressed as mean ± SD. The rest of variables are presented as the number (%)

*ACE*, angiotensin-converting-enzyme; *ARB*, angiotensin II receptor blocker; *CAD*, coronary artery disease

**Table 2:** Baseline vs. 6-Month Follow-up variables

	Baseline	6 months	*P* value
NYHA class			.002
I		61 (35)	
II	51 (26)	75 (43)	
III	114 (58)	32 (18)	
IV	31 (16)	8 (4)	
LVEF (%)	28 (11), (N = 195)	33 (16) (N = 157)	<.001
MLHF score	48 (20) (N = 143)	29 (22) (N = 147)	<.001
LVESV (ml)	191 (94) (N = 198)	160 (116) (N = 156)	<.001

*LVEF*, left ventricular ejection fraction; *LVESV*, left ventricular end-systolic volume; *NYHA*, New York Heart Association

At six-month follow-up, the medications of the 179 living patients were as follows: beta blockers (150 patients, 84%), angiotensin-converting-enzyme inhibitors (105, 59%), statin (58, 32%), aspirin (84, 47%), angiotensin II receptor blockers (43, 24%), diuretics (133, 74%), mineralocorticoids (17, 9%).

### Improvement with CRT

Overall, the primary outcome occurred in 152 out of 179 (84.9%) patients. Functional class improved by at least 1 NYHA class in 67% patients, improvement in LVESV by ≥15% occurred in 54% and LVEF improved by ≥5% in 47%. The MLHF score improved by ≥5 points in 76% of those for whom data were available. Sixteen patients died between the baseline and follow-up period, and thus were excluded from the analysis. Twelve out of 129 (9.3%) patients with LV dyssynchrony, defined as LV PSD > 43°, died compared to four out of 66 (6.1%, *P* = .585).

### Left Ventricular Dyssynchrony and Clinical Outcomes

The mean LV PSD at baseline was 54.4° (minimum 10.3, maximum 113) and PSD was >43° in 129 (66%) patients. Baseline dyssynchrony was not associated with the primary outcome in univariable (OR 0.7, 95% CI 0.33-1.5) or multivariable analyses (OR 0.66, 95% CI 0.25-1.76). The area under the ROC was 0.43. In 46 (23%) patients the CRT lead was placed in the last viable contracting segment, and in 84 (42%), it was placed in either this segment or in a segment that was within 10° of it. Dyssynchrony was not associated with the primary outcome among these patients.

### Change in LV Dyssynchrony with CRT and Clinical Outcomes

The change in LV PSD at six months for the following categories was 10.5° for the overall population, 9.1° for on-target, and 8.8° for on-target/acceptable segments lead placement (not statistically significant from overall). Only nine patients had completely off-target lead placement. There was no significant relationship of an off-target lead placement with the primary outcome. Odds ratios associated with the improvement in LV dyssynchrony and occurrence of the primary outcome are shown in Table 3. Among all study patients, the change in LV PSD from baseline was associated with a small but significant improvement in the primary outcome (OR 1.04, 95% CI 1.01-1.07, *P* = .007; OR = 1.04 per 1° decrease in LV PSD). Patients with on-target (OR 1.55, 95% CI 0.63-3.84) or acceptable lead placement (OR 1.53, 95% CI 0.71-3.28) also were associated with the primary outcome but the relationships were not statistically significant.

**Table 3:** Improvement in LV dyssynchrony and occurrence of the primary outcome

Variable	Odds ratio (95% CI)	
	Unadjusted	Adjusted
Improvement in LV dyssynchrony by SPECT	1.04 (1.01, 1.07)	1.04 (1.01, 1.07), *P* = .007
Age (years)	1.0 (0.98, 1.0)	1.0 (0.95, 1.08), *P* = .744
Females	0.51 (0.25, 1.1)	0.58 (0.12, 2.84), *P* = .502
QRS duration (ms)	1.0 (0.99, 1.0)	1.0 (0.99, 1.02), *P* = .638
NYHA (III & IV) at baseline	0.86 (0.4, 1.9)	2.70 (0.68, 10.77), *P* = .16
LVEF at baseline	1.0 (0.97, 1.0)	0.92 (0.85, 1.00), *P* = .061
LVESV at baseline	0.99 (0.99, 1.0)	0.99 (0.98, 1.0), *P* = .045

*CI*, confidence interval; *LVEF*, left ventricular ejection fraction; *LVESV*, left ventricular end-systolic volume; *NYHA*, New York Heart Association; *SPECT*, single-photon emission computed tomography

**Figure 2:**
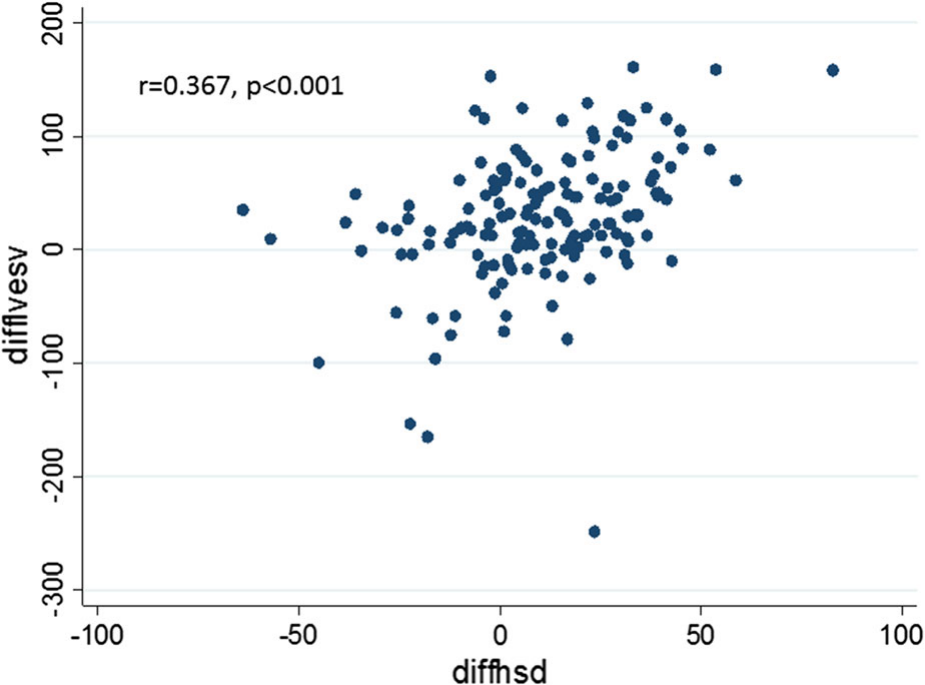
Correlation between phase standard deviation (SD) and end systolic volume (ESV) changes post CRT in all patients. The change in LV PSD correlated with a change in LV ESV from baseline (*r* = 0.367, *P* < .001)

The change in LV PSD correlated with a change in LV ESV from baseline (*r* = .367, *P* < .001) (Figure 2). The position of the lead in relation to the last viable contracting segment, however, did not result in a larger change in ESV (30 mL vs. 28 mL, *P* = .62).

**Figure 3:**
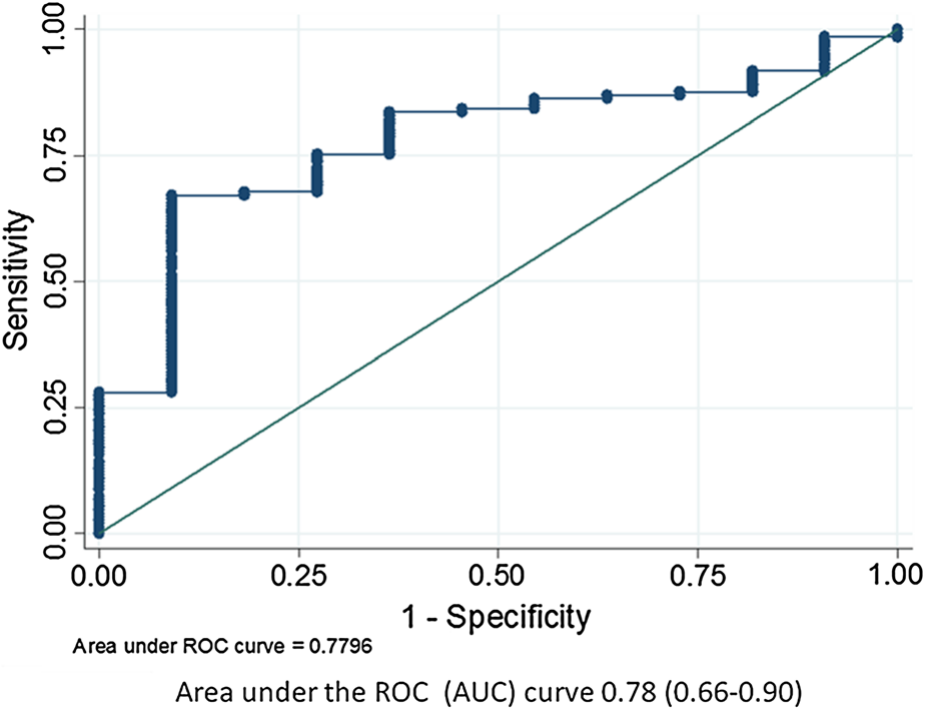
Improvement in LV dyssynchrony with CRT is associated with the primary outcome. The area under the ROC was 0.78 (0.66-0.90) for the association of the improvement in LV PSD with the primary outcome

The area under the ROC was 0.78 (0.66-0.90) for the association of the improvement in LV PSD with the primary outcome (Figure 3). An improvement in LV PSD of 1° was associated with a sensitivity of 71.9% and a specificity of 72.7%. An improvement in LV PSD of 4° was associated with the highest positive likelihood ratio of 7.4 for a favorable clinical outcome. This improvement cutoff was obtained from the ROC analysis in Figure 3.

## Discussion

In this study, we found that baseline LV dyssynchrony was not associated with the primary outcome. However, an improvement in LV PSD of 4° was associated with the highest positive likelihood ratio of 7.4 for a favorable clinical outcome; then changes in LV PSD paralleled changes in the primary outcome and its components, indicating that a dyssynchrony reduction as measured by LV PSD could be associated with outcomes improvement.

The fact that a 4-degree change was significant points to the strength of the discriminatory power of the change in PSD and the fact that there is no need to look for a more robust method. Moreover, the developers of the PSD method used have established that a phase difference of as low as 2.8 degrees can be detected.^31^ Thus, a 4-degree difference is significant when measuring changes in the same patient.

On the other hand, the lead placement in relation to vSOLA on-target or acceptable segments identified by gSPECT MPI did not correlate with clinical outcomes in our study.

### Strengths of the Vision-CRT Study

Our results are based on the study of a contemporary international multicenter population receiving standard state of the art contemporary heart failure therapy, and are comparable in terms of outcome with other heart failure similar populations who received CRT.^19^,^32^-^34^ Thus, our results represent international, multicenter, diverse populations, which reflects everyday clinical practice across these developing countries. Moreover, the software analysis of gSPECT MPI was totally automated and done by a central core lab, and electrophysiologists were not aware of the gSPECT MPI results.

Considering that the post hoc analysis of LV lead positioning in the MADIT-CRT cohort revealed that apical positioning, compared to basal positioning of the LV lead, results in a significant increase in heart failure events and death,^35^ in this study only one patient was paced in apex, and seven patients were paced in non-viable segments.

### Association of LV Dyssynchrony to Clinical Outcomes

It has been shown that the presence of baseline mechanical dyssynchrony measured by echo was associated with a response to resynchronization therapy.^36^ In connection to this, for Delgado et al.,^10^ baseline LV radial dyssynchrony, discordant LV lead position, and myocardial scar in the region of the LV pacing lead were independent determinants of long-term prognosis in ischemic heart failure patients treated with CRT. However, no potential marker of mechanical dyssynchrony reliably predicts response. The lack of predictability is especially evident when different centers attempt to replicate each other’s work, due to the fact that test performance varies widely between centers.^10^ In our case, this possibility does not exist because all baseline and post-CRT studies were processed in a core lab.

On the other hand, Naegeli et al. have shown that after successful CRT implantation, clinical long-term response (27 ± 19 months) is independent of correction of dyssynchrony measured by echocardiography parameters and QRS width. They found several other factors indicating poor outcome such as markers of poor cardiac function, diabetes and poor renal function.^37^

In the present work, we have found that the difference between baseline and six months post-CRT dyssynchrony is a sensitive parameter of clinical outcomes, rather than the baseline value by itself. Thus, LV dyssynchrony automatically measured by PSD from gSPECT MPI is a valid marker of CRT clinical outcomes. This may have an important clinical implication because it represents a sensitive way to predict outcomes to be used in the clinical evaluation of these patients, and as far as we know, our work is the first to give a value of change of LV PSD with a high positive likelihood ratio.

Bleeker et al. reported an acute improvement in LV dyssynchrony immediately after biventricular pacing, and defined this improvement as an acute phenomenon.^36^ Therefore, we consider that rather than using only the baseline dyssynchrony values to predict outcomes, a better approach may be to compare the baseline with the immediate post-CRT dyssynchrony to predict clinical outcomes at six months.

### Association of Recommendation of Lead Placement to Clinical Outcomes

Ideally, the LV lead of a CRT device would be placed at the precise location of latest electromechanical activation in a viable segment. Therefore, LV mechanical dyssynchrony, site of latest mechanical activation, and myocardial scarring are important parameters related to CRT response.^18^,^19^ Theoretically, this positioning would result in optimal resynchronization. By using gSPECT MPI, Boogers et al found that the patients in whom the LV lead was positioned in the latest activated region had a significant response to CRT compared with patients with a discordant LV lead position (79 vs. 26%; *P* < .01).^18^

Nevertheless, we did not find clinical improvement with on-target lead placement, supported by the fact that the degree of dyssynchrony was not significantly reduced in these patients. Two main potential reasons can be advocated: first, we depended on electrophysiologists to report the lead placement site, which could not have been totally exact; second, the algorithm to detect the last viable contracting segment may need further improvement. In addition to this, a third reason could be that the electrophysiologists involved in the study, although blinded to the gSPECT MPI results, received the echo information including the sites of scar if present, and this could have influenced the decision to not pace the non-viable tissue. In fact, only in 3.58% (7/195) of our patients the lead was placed in a hypoperfused non-viable segment as compared to the 19% (17/90) of the patients in the Boogers study.^18^

In contrast to our results, some studies using echocardiography and speckle tracking method,^38^,^39^ have demonstrated that with guided positioning of the lead it is possible to improve the proportion of responders to CRT in heart failure patients. The randomized TARGET^19^ (two-center) trial, with speckle-tracking site provided analysis, found a greater proportion of responders in six months (70% vs. 55%, *P* = .031) when the LV lead was guided to be positioned in vSOLA vs. a control group. Responders were identified as a ≥15% ESV reduction at six months measured by echo. They also found a higher proportion of implanted leads at a scar site 12% (24/207) with twice as many scar implantations in their control group. Moreover, the day after CRT implantation ventricular delays was optimized by echo. This is consistent with our finding that improvement in LV PSD by gSPECT MPI post CRT is a significant predictor of clinical outcomes. However, it must be noted that reliable echocardiographic measurements require expertise in order to obtain consistent and reproducible results. Automated phase analysis from gSPECT MPI has a significant impact clinically because it allows gSPECT MPI, the most widely used nuclear imaging procedure for the management of CAD, to assess cardiac dyssynchrony and heart failure.^40^,^41^

## Limitations of VISION-CRT

The main limitation is that this trial was not designed as a randomized trial. This was necessary in order to obtain international electrophysiologists’ participation to perform implantations at their discretion using their guidelines and not be guided by the gSPECT MPI results. Thus, although all patient recruitment and analysis were done prospectively, the decision as to whether the lead was placed on target or not was determined retrospectively, albeit blinded to all results. Here the patients where the LV lead was not placed in the SPECT recommended sites acted as the control group. Another limitation of the trial is that the location of the LV lead position was provided by the electrophysiologist who placed it and this was not independently verified.

## Conclusions

LV dyssynchrony improvement by gSPECT MPI, but not on-target lead placement, predicts clinical outcomes in patients undergoing CRT. In addition, the fact that PSD change was very significant supports LV PSD as an important physiological variable associated with CRT response and further investigation is required.

## New Knowledge Gained

The fact that the change in PSD is very significant whether the lead was on-target or off-target indicates that LV PSD is an important related physiological variable associated with response to CRT. However, further investigation is required.

## Electronic supplementary material

Below is the link to the electronic supplementary material.

Supplementary material 1 (PPTX 351 kb)

## Abbreviations

CRT: Cardiac resynchronization therapy
CAD: Coronary artery disease
ESV: End-systolic volume
gSPECT MPI: Gated SPECT myocardial perfusion imaging
IAEA: International Atomic Energy Agency
LV: Left ventricle
LVEF: Left ventricular ejection fraction
MLHFQ: Minnesota Living With Heart Failure Questionnaire
NYHA: New York Heart Association
ROC: Receiver-operating curves
SPECT: Single-photon emission computed tomography
vSOLA: Viable segment with the latest mechanical activation

## References

[1] Najafi F, Jamrozik K, Dobson AJ (2009). Understanding the ‘epidemic of heart failure’: A systematic review of trends in determinants of heart failure. Eur J Heart Fail.

[2] Cubillos-Garzon LA, Casas JP, Morillo CA, Bautista LE (2004). Congestive heart failure in Latin America: The next epidemic. Am Heart J.

[3] Leclercq C, Kass DA (2002). Retiming the failing heart: Principles and current clinical status of cardiac resynchronization. J Am Coll Cardiol.

[4] Abraham WT, Hayes DL (2003). Cardiac resynchronization therapy for heart failure. Circulation.

[5] Leclercq C, Hare JM (2004). Ventricular resynchronization: Current state of the art. Circulation.

[6] Leclercq C, Faris O, Tunin R, Johnson J, Kato R, Evans F (2002). Systolic improvement and mechanical resynchronization does not require electrical synchrony in the dilated failing heart with left bundle-branch block. Circulation.

[7] Achilli A, Sassara M, Ficili S, Pontillo D, Achilli P, Alessi C (2003). Long-term effectiveness of cardiac resynchronization therapy in patients with refractory heart failure and “narrow” QRS. J Am Coll Cardiol.

[8] Sillanmaki S, Lipponen JA, Tarvainen MP, Laitinen T, Hedman M, Hedman A et al. Relationships between electrical and mechanical dyssynchrony in patients with left bundle branch block and healthy controls. J Nucl Cardiol 2018. 10.1007/s12350-018-1204-0. 10.1007/s12350-018-1204-0 29423906

[9] Bax JJ, Bleeker GB, Marwick TH, Molhoek SG, Boersma E, Steendijk P (2004). Left ventricular dyssynchrony predicts response and prognosis after cardiac resynchronization therapy. J Am Coll Cardiol.

[10] Delgado V, van Bommel RJ, Bertini M, Borleffs CJ, Marsan NA, Arnold CT (2011). Relative merits of left ventricular dyssynchrony, left ventricular lead position, and myocardial scar to predict long-term survival of ischemic heart failure patients undergoing cardiac resynchronization therapy. Circulation.

[11] Bax JJ, Abraham T, Barold SS, Breithardt OA, Fung JW, Garrigue S (2005). Cardiac resynchronization therapy: Part 1—Issues before device implantation. J Am Coll Cardiol.

[12] Tanaka H, Nesser HJ, Buck T, Oyenuga O, Jánosi RA, Winter S (2010). Dyssynchrony by speckle-tracking echocardiography and response to cardiac resynchronization therapy: Results of the Speckle Tracking and Resynchronization (STAR) study. Eur Heart J.

[13] Leyva F (2017). The role of cardiovascular magnetic resonance in cardiac resynchronization therapy. Heart Fail Clin.

[14] Somsen GA, Verberne HJ, Burri H, Ratib O, Righetti A (2006). Ventricular mechanical dyssynchrony and resynchronization therapy in heart failure: A new indication for Fourier analysis of gated blood-pool radionuclide ventriculography. Nucl Med Commun.

[15] Badhwar N, James J, Hoffmayer KS, O’Connell JW, Green D, De Marco T (2016). Utility of equilibrium radionuclide angiogram-derived measures of dyssynchrony to predict outcomes in heart failure patients undergoing cardiac resynchronization therapy. J Nucl Med.

[16] Chen J, Garcia EV, Folks RD, Cooke CD, Faber TL, Tauxe EL (2005). Onset of left ventricular mechanical contraction as determined by phase analysis of ECG-gated myocardial perfusion SPECT imaging: Development of a diagnostic tool for assessment of cardiac mechanical dyssynchrony. J Nucl Cardiol.

[17] Van Kriekinge SD, Nishina H, Ohba M, Berman DS, Germano G (2008). Automatic global and regional phase analysis from gated myocardial perfusion SPECT imaging: Application to the characterization of ventricular contraction in patients with left bundle branch block. J Nucl Med.

[18] Boogers MJ, Chen J, van Bommel RJ, Borleffs CJ, Dibbets-Schneider P, van der Hiel B (2011). Optimal left ventricular lead position assessed with phase analysis on gated myocardial perfusion SPECT. Eur J Nucl Med Mol Imaging.

[19] Khan FZ, Virdee MS, Palmer CR, Pugh PJ, O’Halloran D, Elsik M (2012). Targeted left ventricular lead placement to guide cardiac resynchronization therapy: The TARGET study: A randomized, controlled trial. J Am Coll Cardiol.

[20] Friehling M, Chen J, Saba S, Bazaz R, Schwartzman D, Adelstein EC (2011). A prospective pilot study to evaluate the relationship between acute change in left ventricular synchrony after cardiac resynchronization therapy and patient outcome using a single-injection gated SPECT protocol. Circ Cardiovasc Imaging.

[21] Henzlova MJ, Duvall WL, Einstein AJ, Travin MI, Verberne HJ (2016). ASNC imaging guidelines for SPECT nuclear cardiology procedures: Stress, protocols, and tracers. J Nucl Cardiol.

[22] Trimble MA, Borges-Neto S, Honeycutt EF, Shaw LK, Pagnanelli R, Chen J (2008). Evaluation of mechanical dyssynchrony and myocardial perfusion using phase analysis of gated SPECT imaging in patients with left ventricular dysfunction. J Nucl Cardiol.

[23] Trimble MA, Velazquez EJ, Adams GL, Honeycutt EF, Pagnanelli RA, Barnhart HX (2008). Repeatability and reproducibility of phase analysis of gated single-photon emission computed tomography myocardial perfusion imaging used to quantify cardiac dyssynchrony. Nucl Med Commun.

[24] Lin X, Xu H, Zhao X, Folks RD, Garcia EV, Soman P (2010). Repeatability of left ventricular dyssynchrony and function parameters in serial gated myocardial perfusion SPECT studies. J Nucl Cardiol.

[25] Pazhenkottil AP, Buechel RR, Herzog BA, Nkoulou RN, Valenta I, Fehlmann U (2010). Ultrafast assessment of left ventricular dyssynchrony from nuclear myocardial perfusion imaging on a new high-speed gamma camera. Eur J Nucl Med Mol Imaging.

[26] Aljaroudi W, Koneru J, Heo J, Iskandrian AE (2011). Impact of ischemia on left ventricular dyssynchrony by phase analysis of gated single photon emission computed tomography myocardial perfusion imaging. J Nucl Cardiol.

[27] Cheung A, Zhou Y, Faber TL, Garcia EV, Zhu L, Chen J (2012). The performance of phase analysis of gated SPECT myocardial perfusion imaging in the presence of perfusion defects: A simulation study. J Nucl Cardiol.

[28] Zhou W, Tao N, Hou X, Wang Y, Folks RD, Cooke DC et al. Development and validation of an automatic method to detect the latest contracting viable left ventricular segments to assist guide CRT therapy from gated SPECT myocardial perfusion imaging. J Nucl Cardiol 2017. 10.1007/s12350-017-0853-8. 10.1007/s12350-017-0853-8 PMC10981925 28353213

[29] Zhou W, Garcia EV (2016). Nuclear image-guided approaches for cardiac resynchronization therapy (CRT). Curr Cardiol Rep.

[30] Henneman MM, Chen J, Ypenburg C, Dibbets P, Bleeker GB, Boersma E (2007). Phase analysis of gated myocardial perfusion single-photon emission computed tomography compared with tissue Doppler imaging for the assessment of left ventricular dyssynchrony. J Am Coll Cardiol.

[31] Chen J, Faber TL, Cooke CD, Garcia EV (2008). Temporal resolution of multiharmonic phase analysis of ECG-gated myocardial perfusion SPECT studies. J Nucl Cardiol.

[32] Mullens W, Tang WH (2011). Optimizing cardiac resynchronization therapy in advanced heart failure. Congest Heart Fail.

[33] Hoke U, Bax JJ, Delgado V, Ajmone Marsan N (2018). Assessment of left ventricular dyssynchrony by three-dimensional echocardiography: Prognostic value in patients undergoing cardiac resynchronization therapy. J Cardiovasc Electrophysiol..

[34] Vidula H, Kutyifa V, McNitt S, Goldenberg I, Solomon SD, Moss AJ (2017). Long-term survival of patients with left bundle branch block who are hypo-responders to cardiac resynchronization therapy. Am J Cardiol.

[35] Singh JP, Klein HU, Huang DT, Reek S, Kuniss M, Quesada A (2011). Left ventricular lead position and clinical outcome in the multicenter automatic defibrillator implantation trial-cardiac resynchronization therapy (MADIT-CRT) trial. Circulation.

[36] Bleeker GB, Mollema SA, Holman ER, Van de Veire N, Ypenburg C, Boersma E (2007). Left ventricular resynchronization is mandatory for response to cardiac resynchronization therapy: Analysis in patients with echocardiographic evidence of left ventricular dyssynchrony at baseline. Circulation.

[37] Naegeli B, Brunner-La Rocca HP, Attenhofer Jost C, Fah-Gunz A, Maurer D, Bertel O (2014). Clinical long-term response to cardiac resynchronization therapy is independent of persisting echocardiographic markers of dyssynchrony. Cardiol Res.

[38] Mele D, Bertini M, Malagu M, Nardozza M, Ferrari R (2017). Current role of echocardiography in cardiac resynchronization therapy. Heart Fail Rev.

[39] Mele D, Nardozza M, Malagu M, Leonetti E, Fragale C, Rondinella A (2017). Left ventricular lead position guided by parametric strain echocardiography improves response to cardiac resynchronization therapy. J Am Soc Echocardiogr.

[40] Chen J, Henneman MM, Trimble MA, Bax JJ, Borges-Neto S, Iskandrian AE (2008). Assessment of left ventricular mechanical dyssynchrony by phase analysis of ECG-gated SPECT myocardial perfusion imaging. J Nucl Cardiol.

[41] Chen J, Garcia EV, Bax JJ, Iskandrian AE, Borges-Neto S, Soman P (2011). SPECT myocardial perfusion imaging for the assessment of left ventricular mechanical dyssynchrony. J Nucl Cardiol.

