# Supplementary material for: Value of intraventricular dyssynchrony assessment by gated-SPECT myocardial perfusion imaging in the management of heart failure patients undergoing cardiac resynchronization therapy (VISION-CRT)
Source: J Nucl Cardiol. 2019 Jan 25;28(1):55–64. doi: 10.1007/s12350-018-01589-5 (PMC7921049; doi:10.1007/s12350-018-01589-5)
Supplement: Supplementary file 1 — Supplementary material 1 (PPTX 351 kb) [file 12350_2018_1589_MOESM1_ESM.pptx]

## Slide 1
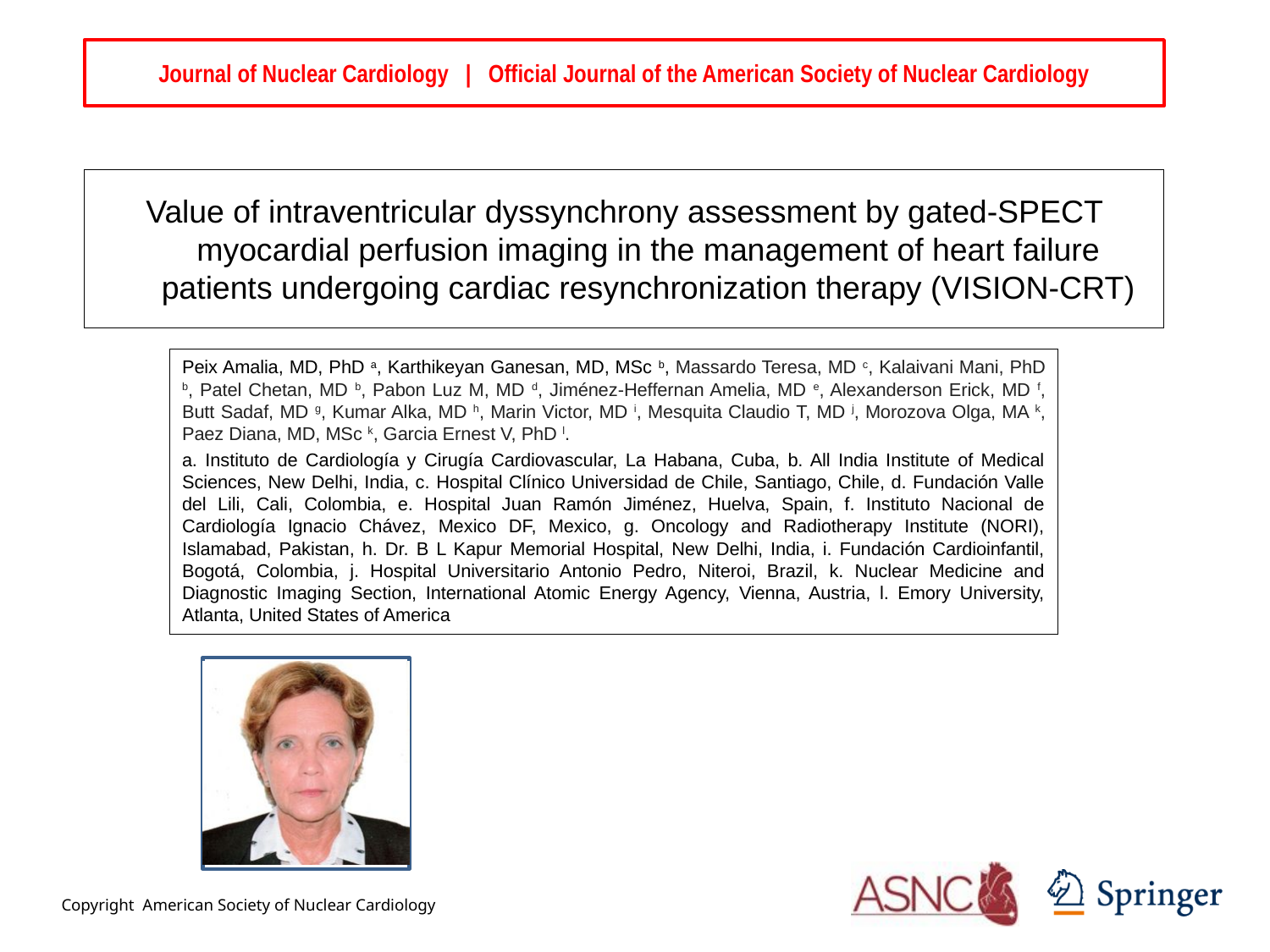

Journal of Nuclear Cardiology | Official Journal of the American Society of Nuclear Cardiology
# Value of intraventricular dyssynchrony assessment by gated-SPECT myocardial perfusion imaging in the management of heart failure patients undergoing cardiac resynchronization therapy (VISION-CRT)
Peix Amalia, MD, PhD a, Karthikeyan Ganesan, MD, MSc b, Massardo Teresa, MD c, Kalaivani Mani, PhD b, Patel Chetan, MD b, Pabon Luz M, MD d, Jiménez-Heffernan Amelia, MD e, Alexanderson Erick, MD f, Butt Sadaf, MD g, Kumar Alka, MD h, Marin Victor, MD i, Mesquita Claudio T, MD j, Morozova Olga, MA k, Paez Diana, MD, MSc k, Garcia Ernest V, PhD l.
a. Instituto de Cardiología y Cirugía Cardiovascular, La Habana, Cuba, b. All India Institute of Medical Sciences, New Delhi, India, c. Hospital Clínico Universidad de Chile, Santiago, Chile, d. Fundación Valle del Lili, Cali, Colombia, e. Hospital Juan Ramón Jiménez, Huelva, Spain, f. Instituto Nacional de Cardiología Ignacio Chávez, Mexico DF, Mexico, g. Oncology and Radiotherapy Institute (NORI), Islamabad, Pakistan, h. Dr. B L Kapur Memorial Hospital, New Delhi, India, i. Fundación Cardioinfantil, Bogotá, Colombia, j. Hospital Universitario Antonio Pedro, Niteroi, Brazil, k. Nuclear Medicine and Diagnostic Imaging Section, International Atomic Energy Agency, Vienna, Austria, l. Emory University, Atlanta, United States of America
Head shot of author
required
Copyright American Society of Nuclear Cardiology

## Slide 2
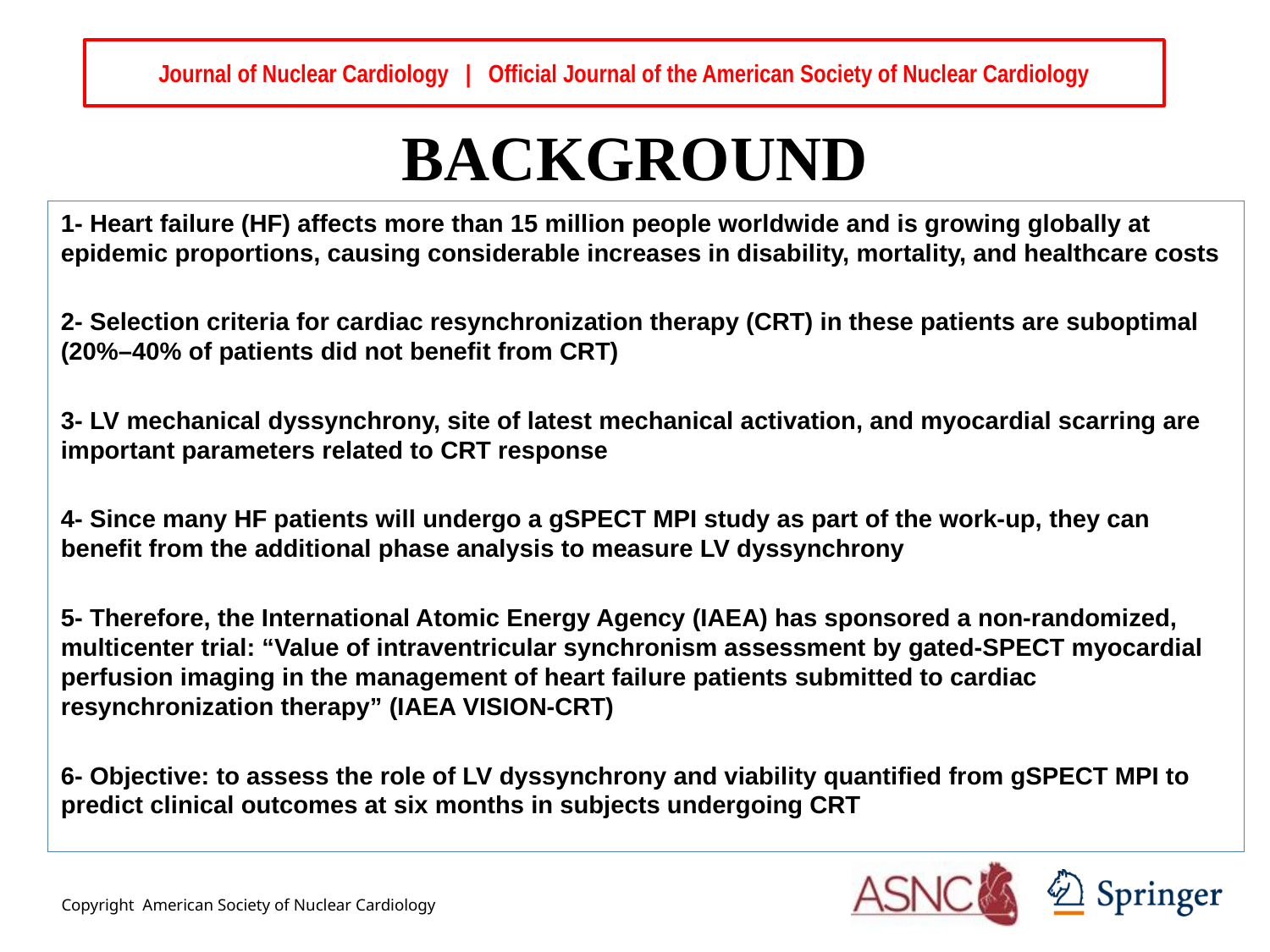

Journal of Nuclear Cardiology | Official Journal of the American Society of Nuclear Cardiology
# BACKGROUND
1- Heart failure (HF) affects more than 15 million people worldwide and is growing globally at epidemic proportions, causing considerable increases in disability, mortality, and healthcare costs
2- Selection criteria for cardiac resynchronization therapy (CRT) in these patients are suboptimal (20%–40% of patients did not benefit from CRT)
3- LV mechanical dyssynchrony, site of latest mechanical activation, and myocardial scarring are important parameters related to CRT response
4- Since many HF patients will undergo a gSPECT MPI study as part of the work-up, they can benefit from the additional phase analysis to measure LV dyssynchrony
5- Therefore, the International Atomic Energy Agency (IAEA) has sponsored a non-randomized, multicenter trial: “Value of intraventricular synchronism assessment by gated-SPECT myocardial perfusion imaging in the management of heart failure patients submitted to cardiac resynchronization therapy” (IAEA VISION-CRT)
6- Objective: to assess the role of LV dyssynchrony and viability quantified from gSPECT MPI to predict clinical outcomes at six months in subjects undergoing CRT
Copyright American Society of Nuclear Cardiology

## Slide 3
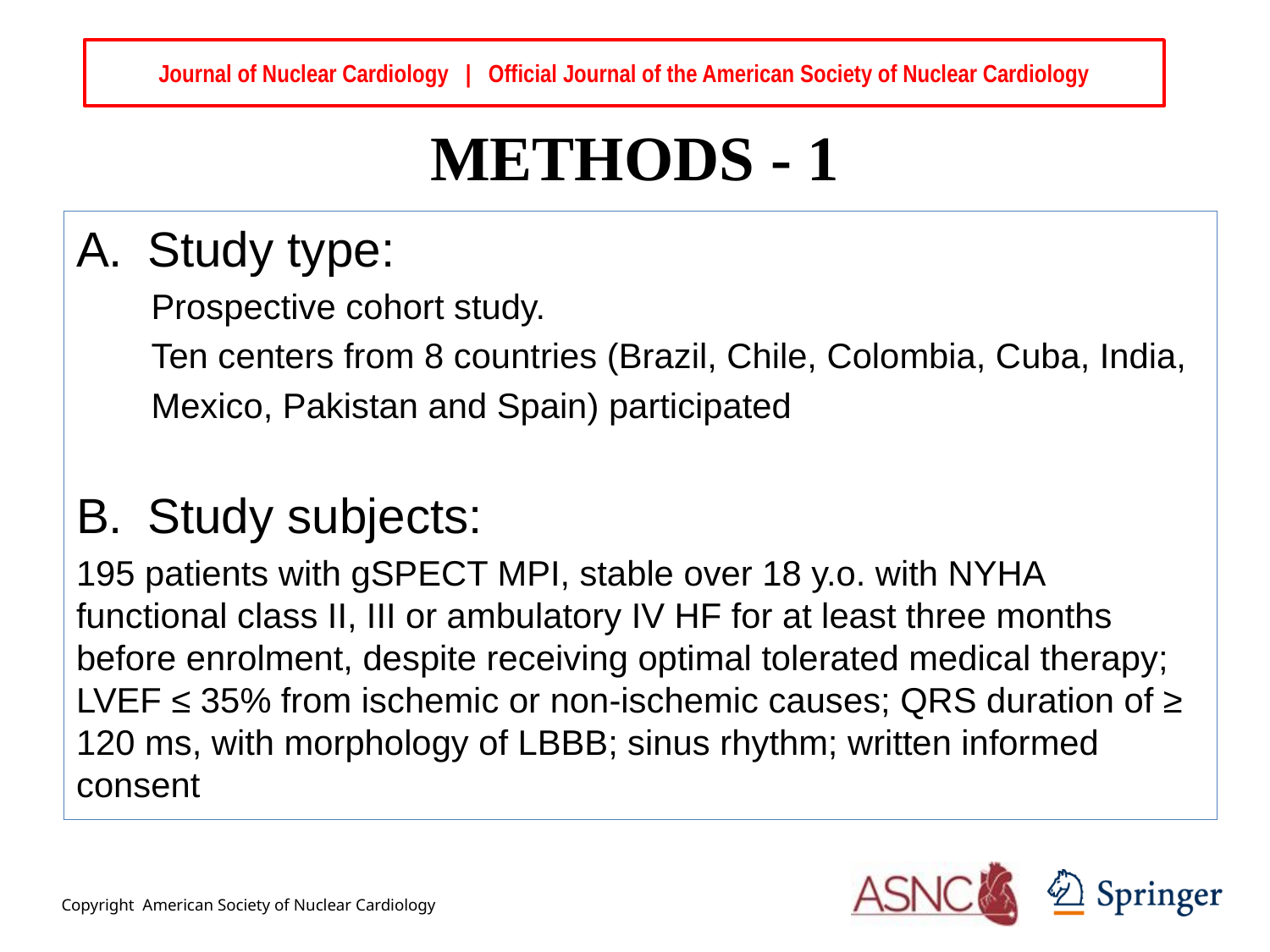

Journal of Nuclear Cardiology | Official Journal of the American Society of Nuclear Cardiology
# METHODS - 1
Study type:
 Prospective cohort study.
 Ten centers from 8 countries (Brazil, Chile, Colombia, Cuba, India,
 Mexico, Pakistan and Spain) participated
Study subjects:
195 patients with gSPECT MPI, stable over 18 y.o. with NYHA functional class II, III or ambulatory IV HF for at least three months before enrolment, despite receiving optimal tolerated medical therapy; LVEF ≤ 35% from ischemic or non-ischemic causes; QRS duration of ≥ 120 ms, with morphology of LBBB; sinus rhythm; written informed consent
Copyright American Society of Nuclear Cardiology

## Slide 4
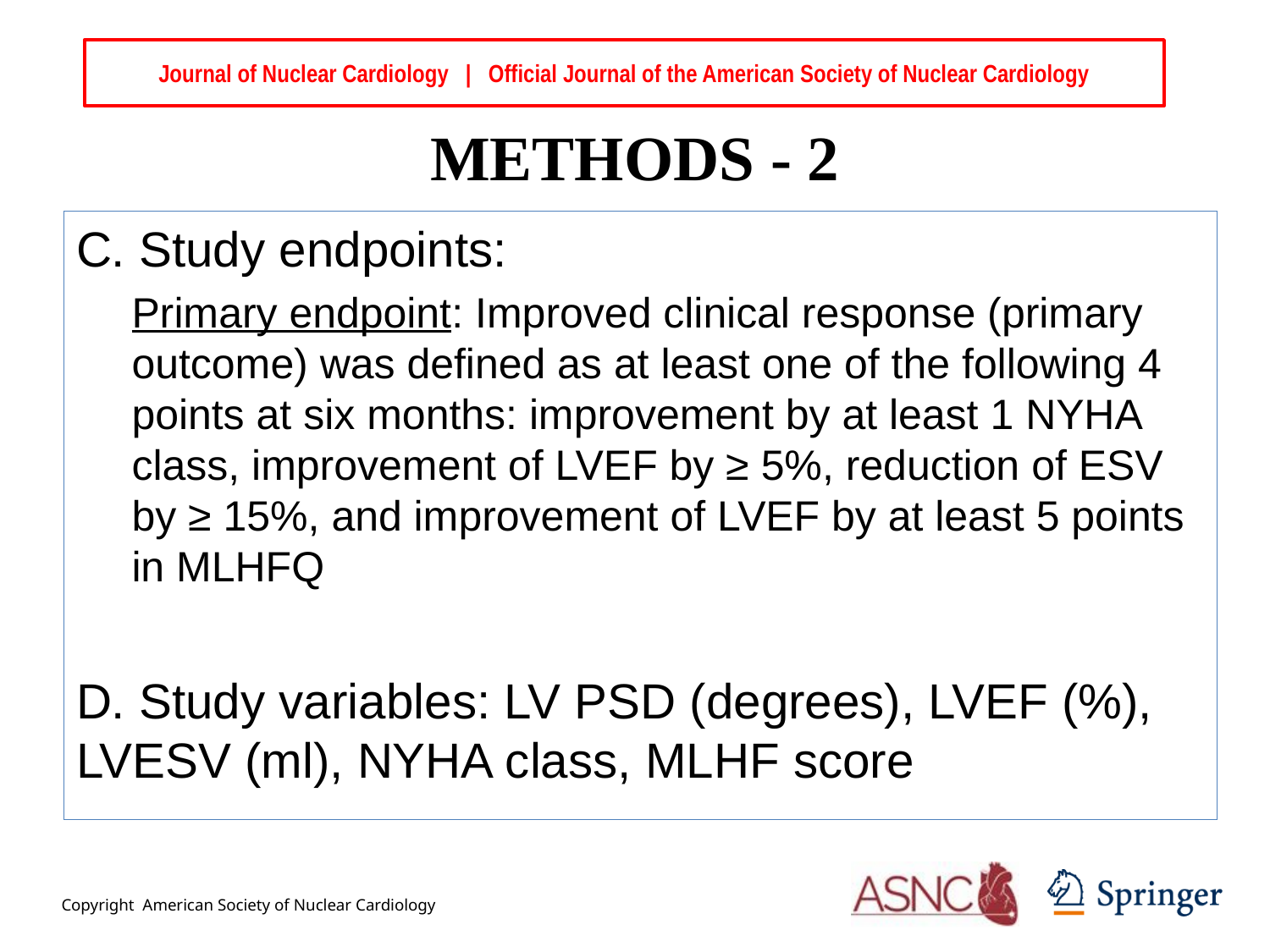

Journal of Nuclear Cardiology | Official Journal of the American Society of Nuclear Cardiology
# METHODS - 2
C. Study endpoints:
Primary endpoint: Improved clinical response (primary outcome) was defined as at least one of the following 4 points at six months: improvement by at least 1 NYHA class, improvement of LVEF by ≥ 5%, reduction of ESV by ≥ 15%, and improvement of LVEF by at least 5 points in MLHFQ
D. Study variables: LV PSD (degrees), LVEF (%), LVESV (ml), NYHA class, MLHF score
Copyright American Society of Nuclear Cardiology

## Slide 5
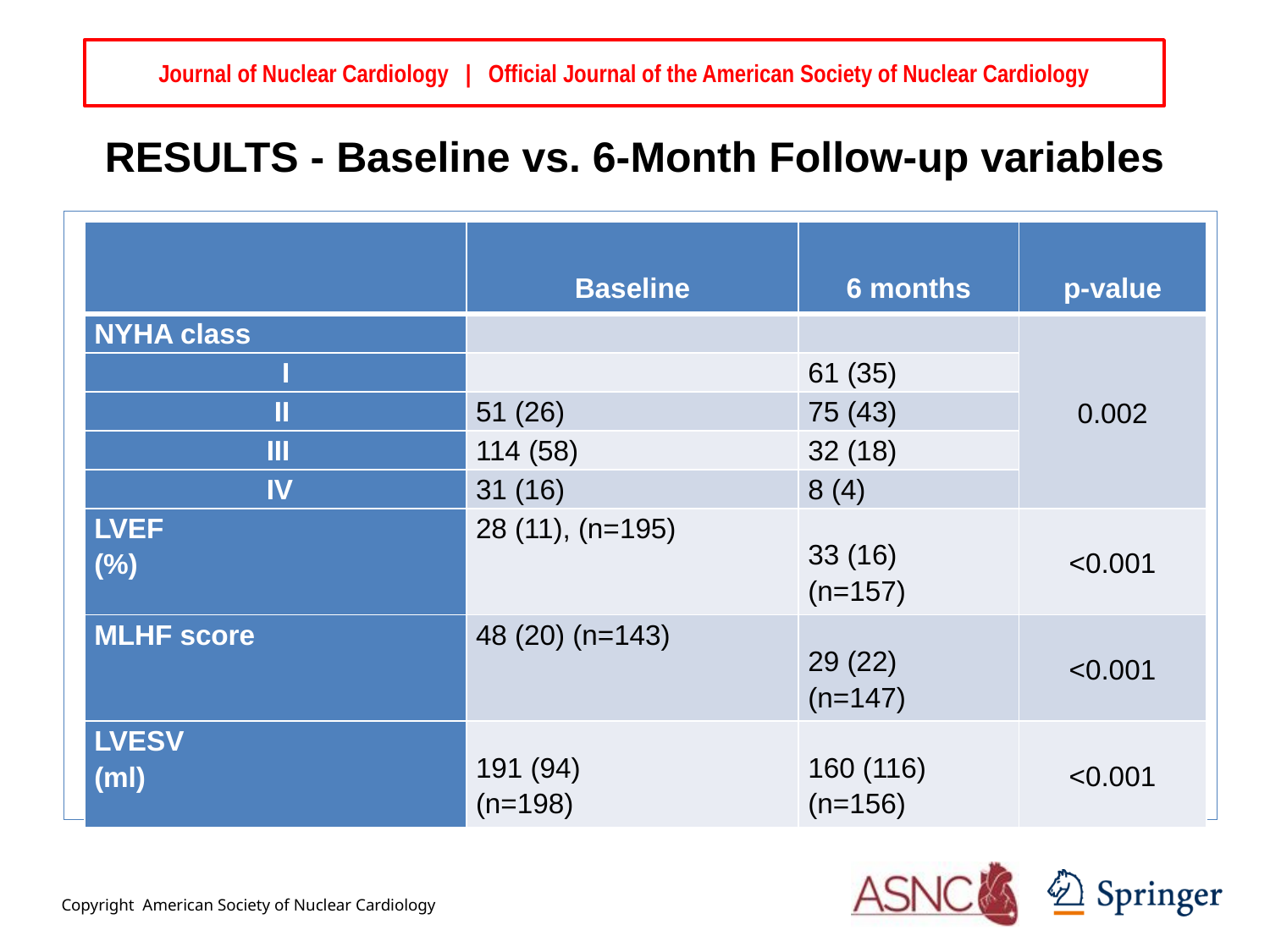

Journal of Nuclear Cardiology | Official Journal of the American Society of Nuclear Cardiology
# RESULTS - Baseline vs. 6-Month Follow-up variables
| | Baseline | 6 months | p-value |
| --- | --- | --- | --- |
| NYHA class | | | 0.002 |
| I | | 61 (35) | |
| II | 51 (26) | 75 (43) | |
| III | 114 (58) | 32 (18) | |
| IV | 31 (16) | 8 (4) | |
| LVEF (%) | 28 (11), (n=195) | 33 (16) (n=157) | <0.001 |
| MLHF score | 48 (20) (n=143) | 29 (22) (n=147) | <0.001 |
| LVESV (ml) | 191 (94) (n=198) | 160 (116) (n=156) | <0.001 |
Copyright American Society of Nuclear Cardiology

## Slide 6
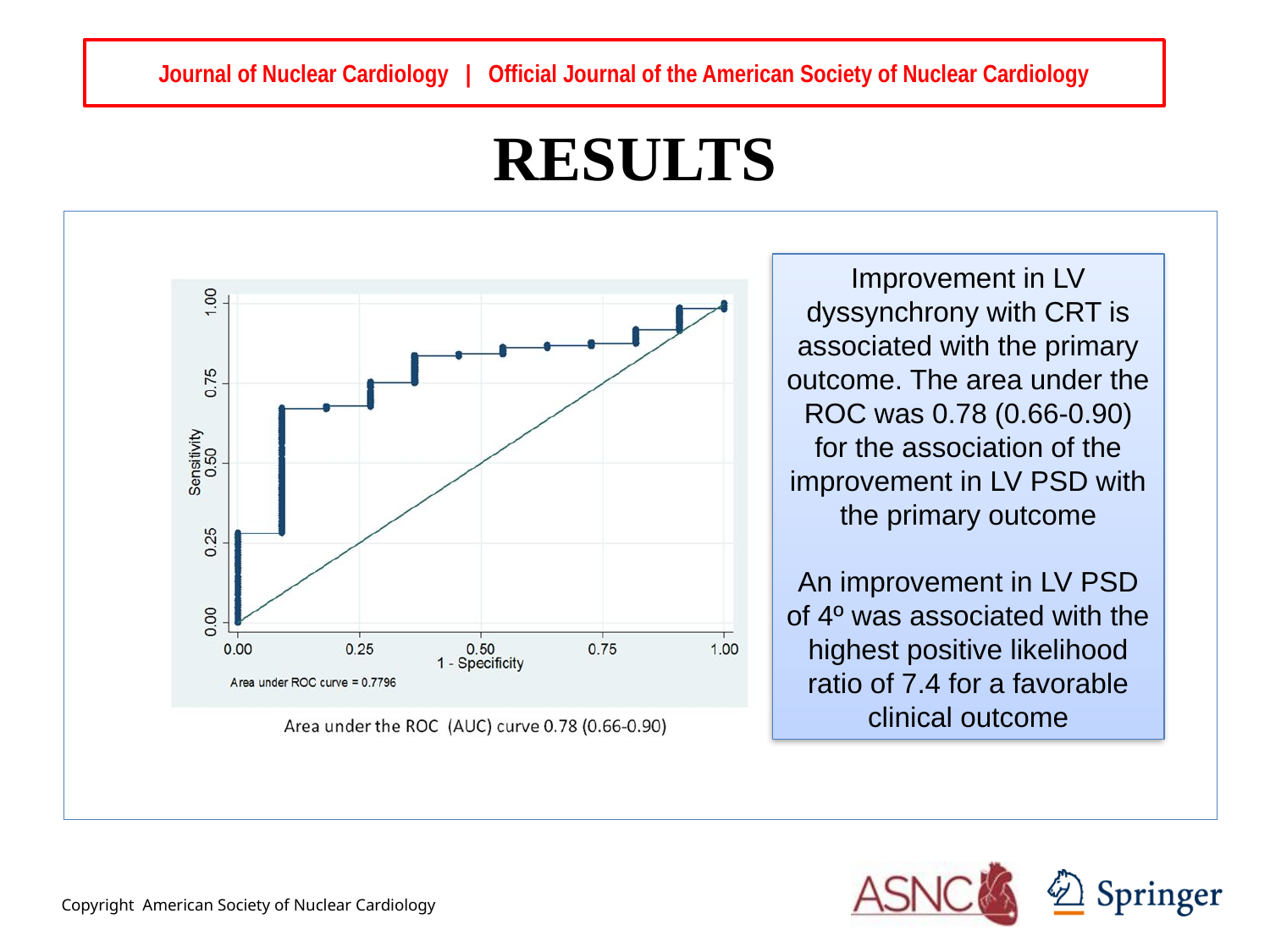

Journal of Nuclear Cardiology | Official Journal of the American Society of Nuclear Cardiology
# RESULTS
Improvement in LV dyssynchrony with CRT is associated with the primary outcome. The area under the ROC was 0.78 (0.66-0.90) for the association of the improvement in LV PSD with the primary outcome
An improvement in LV PSD of 4º was associated with the highest positive likelihood ratio of 7.4 for a favorable clinical outcome
Copyright American Society of Nuclear Cardiology

## Slide 7
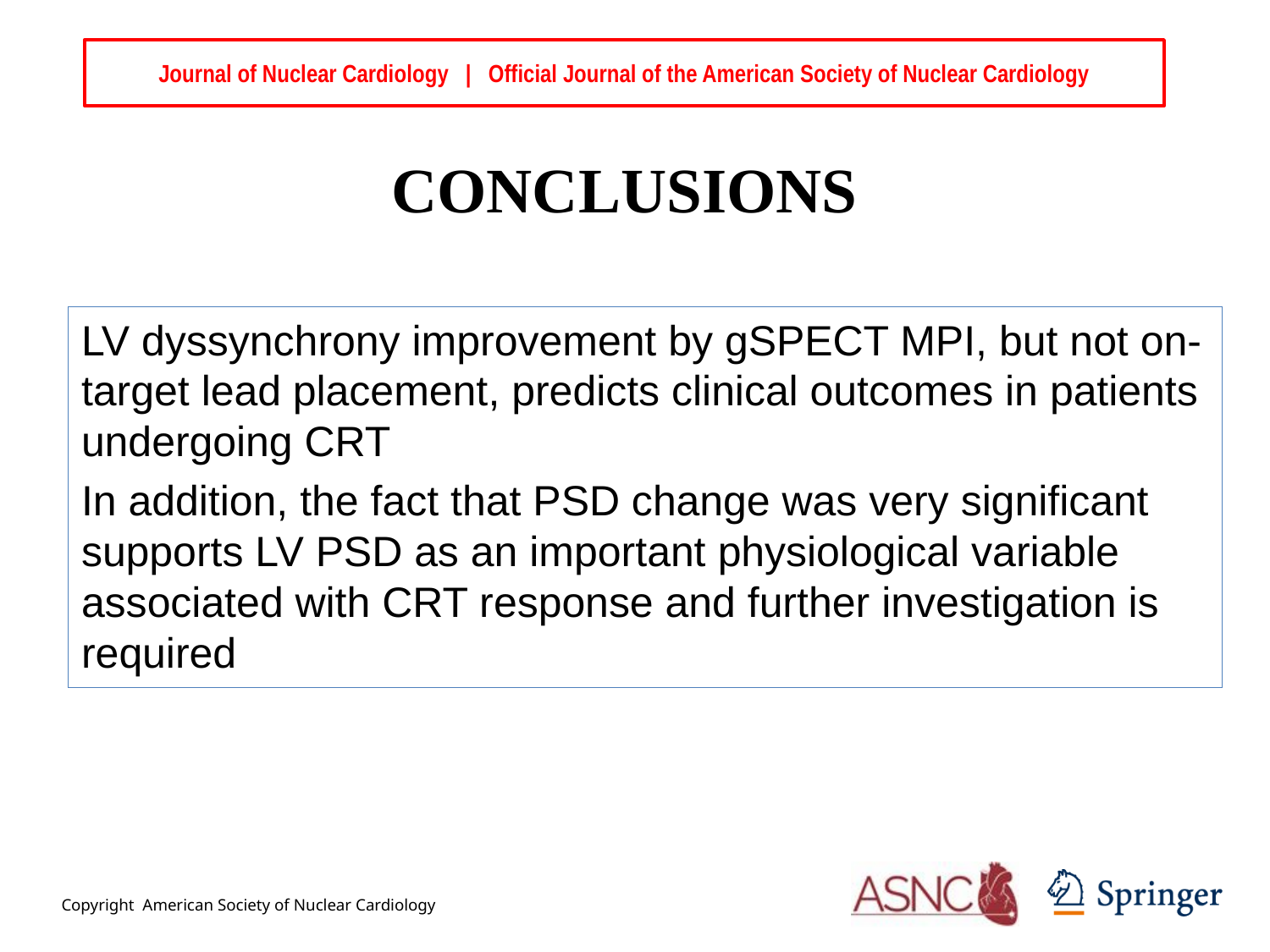

Journal of Nuclear Cardiology | Official Journal of the American Society of Nuclear Cardiology
# CONCLUSIONS
LV dyssynchrony improvement by gSPECT MPI, but not on-target lead placement, predicts clinical outcomes in patients undergoing CRT
In addition, the fact that PSD change was very significant supports LV PSD as an important physiological variable associated with CRT response and further investigation is required
Copyright American Society of Nuclear Cardiology
